# Dually Sulphophilic Chromium Boride Nanocatalyst Boosting Sulfur Conversion Kinetics Toward High‐Performance Lithium–Sulfur Batteries

**DOI:** 10.1002/advs.202303830

**Published:** 2023-09-25

**Authors:** Hongyang Li, Guxian Chen, Kailong Zhang, Liangbiao Wang, Gaoran Li

**Affiliations:** ^1^ MIIT Key Laboratory of Advanced Display Materials and Devices School of Materials Science and Engineering Nanjing University of Science and Technology Nanjing Jiangsu 210094 P. R. China; ^2^ Key Laboratory for Palygorskite Science and Applied Technology of Jiangsu National & Local Joint Engineering Research Center for Mineral Salt Deep Utilization School of Chemical Engineering Huaiyin Institute of Technology Huaian Jiangsu 223003 P. R. China; ^3^ School of Chemistry and Chemical Engineering Jiangsu University of Technology Changzhou Jiangsu 213001 P. R. China

**Keywords:** chromium boride, dual sulphophilicity, electrocatalysis, lithium‐sulfur batteries

## Abstract

The sluggish kinetics of sulfur conversions have long been hindering the implementation of fast and efficient sulfur electrochemistry in lithium–sulfur (Li–S) batteries. In this regard, herein the unique chromium boride (CrB) is developed via a well‐confined mild‐temperature thermal reaction to serve as an advanced sulfur electrocatalyst. Its interstitial‐alloy nature features excellent conductivity, while the nano‐lamination architecture affords abundant active sites for host‐guest interactions. More importantly, the CrB nanocatalyst demonstrates a dual sulphophilicity with simultaneous Cr─S and B─S bondage for establishing strong interactions with the intermediate polysulfides. As a result, significant stabilization and promotion of sulfur redox behavior can be achieved, enabling an excellent Li–S cell cyclability with a minimum capacity fading rate of 0.0176% per cycle over 2000 cycles and a favorable rate capability up to 7 C. Additionally, a high areal capacity of 5.2 mAh cm^−2^, and decent cycling and rate performances are still attainable under high sulfur loading and low electrolyte dosage. This work offers a facile approach and instructive insights into metal boride sulfur electrocatalyst, holding a good promise for pursuing high‐efficiency sulfur electrochemistry and high‐performance Li–S batteries.

## Graphical abstract

Chromium boride is developed for the first time as sulfur electrocatalyst, which imposes a unique dual‐sulphophilic mechanism via p‐d and p‐p hybridizations with the polysulfide intermediates. As a result, fast and durable sulfur electrochemistry is realized, contributing to excellent cyclability, rate capability, and high‐loading performances of Li–S batteries.

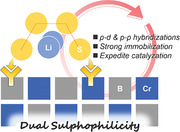

## 1 Introduction

The global target of carbon neutralization necessitates the active development and deployment of green energies such as solar, wind, and hydro.^[^ ^1^ ^]^ Electrochemical energy storage hereto affords an attractive solution to the efficient and convenient utilization of these sustainable but intermittent energies, and has attracted tremendous research enthusiasm during the past few decades.^[^ ^2^ ^]^ The currently mainstream lithium‐ion battery (LIB) technology plays a critical role in electric vehicles and grid energy storage due to its high energy, durability, and technical maturity. However, energy density and affordability bottlenecks increasingly challenge their future implementation in these fields.^[^ ^3^ ^]^ In this context, lithium–sulfur (Li–S) batteries emerge as a promising next‐generation energy storage system attributed to their theoretically high energy density, almost five times that of the conventional LIB (2600 vs 500 Wh kg^−1^), as well as the low‐cost that nearly half‐cut that of LIBs benefiting from the abundant reserve and wide geometrical distribution of sulfur.^[^ ^4^ ^]^

Despite these intriguing merits, Li–S batteries struggle with several critical and characteristic challenges that obstruct their entrance into the practical markets.^[^ ^5^ ^]^ Specifically, the insulating nature of sulfur and sulfides, the multi‐electron transfer reaction pathway, and the liquid‐solid phase‐conversion mechanism collectively determine the intrinsic sluggish kinetics of sulfur reactions, rendering the difficult realization of fast and efficient sulfur electrochemistry. Meanwhile, the high solubility and mobility of the intermediate lithium polysulfides (LiPS) trigger the notorious shuttle effect accompanied by persistent sulfur loss, coulombic inefficiency, and anode passivation, resulting in fast capacity fading and battery failure.^[^ ^6^ ^]^

Given these intractable problems, catalytic agents are desired to stabilize and promote sulfur conversions. In the past few years, researchers worldwide have devoted significant efforts to developing advanced sulfur electrocatalysts for alleviating the kinetic limits and inhibiting shuttle behaviors.^[^ ^7^ ^]^ Among them, chemically polar inorganics such as metal compounds have attracted particular attention due to their strong interactions with LiPS, which are expected to chemically immobilize sulfur species and facilitate their electrochemical conversions.^[^ ^8^ ^]^ Liang et al. presented unique VO_x_ hollow spheres, as representative metal oxide, for chemically adsorbing LiPS following a “Goldilocks” principle to resist the shuttling behavior.^[^ ^9^ ^]^ Cui's group systematically studied the catalytic oxidation of Li_2_S on different polar sulfide surfaces, demonstrating that VS_2_, TiS_2_, and CoS_2_ deliver relatively superior catalytic activity due to their strong interaction with LiPS and the great facilitation of Li‐ion transport.^[^ ^10^ ^]^ Apart from that, metal‐organic frameworks (MOFs) are also potential host matrixes for improving sulfur electrochemistry. Huang's group recently reported an ultralight electroconductive Ni‐TABQ, which efficiently promotes the multistep sulfur conversions benefiting from the dual catalytic centers within the framework, i.e., Ni‐N4 and quinone.^[^ ^11^ ^]^

Despite the encouraging progress, common metal compounds encounter some substantial shortcomings, represented by their poor electrical conductivity and limited access to the active sites, struggling to boost battery performance further. Given this, metal borides (MBs) recently become a rising star in this field due to their intrinsically high conductivity and unique chemical structures.^[^ ^12^ ^]^ Metal‐rich MB with alloy lattice structure inherits good metallicity, endowing high electrical conductivity up to 10^7^ S m^−1^. Additionally, MBs deliver a controllable transition from superconductor to semiconductor and further into insulator along with the increase of metal‐to‐boron ratio in stoichiometry, enabling tailorable chemical and electronic structures that afford attractive opportunities to meet specific demands in extensive areas.^[^ ^13^ ^]^ Given the challenges in the Li–S system, MBs have shown considerable promise in regulating sulfur electrochemistry. Qian's group pioneered a layer‐structure and highly conductive titanium diboride (TiB_2_) as the polar sulfur host material in Li–S batteries.^[^ ^14^ ^]^ A surface passivation mechanism was proposed for enhancing the polysulfide anchoring effect of TiB_2_, thus contributing to a strong sulfur immobilization with stable cell cycling. Li et al. compared TiB_2_ with other Ti‐based compounds, i.e., TiO_2_ and TiC, and revealed the highest LiPS adsorption strength by boride that mostly favors the catalytic sulfur conversions.^[^ ^15^ ^]^ Recently, MoB was also explored as an electrocatalyst for sulfur electrochemistry.^[^ ^16^ ^]^ Combining high conductivity, rich catalytic sites, and good hydrophilicity, the MoB catalyst enables a capacity of 1253 mAh g^−1^, a long lifespan of over 1000 cycles, and reliable sulfur redox under high sulfur loading and limited electrolyte. These advances have demonstrated the great potential of MBs for catalyzing sulfur conversions and improving Li–S battery performance. However, this family of electrocatalysts is still in its infancy, with their structural potentialities to be further exploited, nanostructure to be rationally designed, and underlying functioning mechanisms to be deeply probed into.

In this contribution, chromium boride (CrB) nanocatalyst was developed for the first time to power superior Li–S battery chemistry. The CrB catalyst with unique laminated nanostructure was prepared via a well‐confined, mild‐temperature, and solid‐state thermal method. Benefiting from its 2D interstitial‐alloy lattice structure, CrB inherits good metallicity with a high conductivity of 357 S m^−1^. More importantly, the unique dual‐sulphophilic character of CrB imposes simultaneous p‐d and p‐p hybridizations with LiPS, which is distinct from the Lewis acid‐base mode in conventional catalyst scenarios, and enables strong sulfur immobilization against the shuttle effect and lowered energy barriers for multistep sulfur conversions. As a result, fast, efficient, and durable sulfur redox electrochemistry was realized, instantiated by the excellent cyclability over 2000 cycles, superb rate capability up to 7 C, and decent energy output even under the high‐loading (sulfur loading = 5.0 mg cm^−2^), and lean‐electrolyte (E/S ratio = 5.5 mL g^−1^).

## 2 Results and Discussion

The CrB nanocatalyst was prepared via a well‐confined, mild‐temperature, solid‐state method. CrO_3_ was utilized as the chromium source, while NaBH_4_ was simultaneously the boron source and reductive agent. The as‐built high‐pressure and highly reductive environment enables the mild‐temperature boronization to yield the well‐dispersed nanometric CrB. It should be noted that the one‐pot synthesis with the simple setup and mild conditions endows this method with good scalability for potential practical implementation.

**Figure 1:**
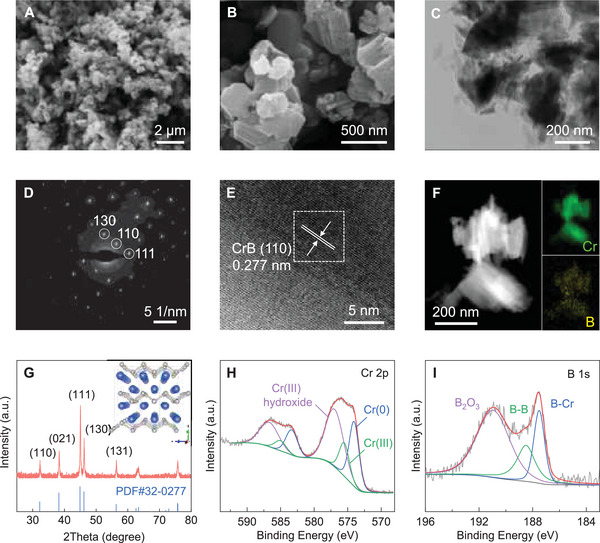
A,B) SEM images, C) TEM image, D) SAED pattern, E) HRTEM image, F) HADDF‐STEM image and elemental mapping, G) XRD pattern and projected crystalline structure (inset), high‐resolution XPS H) Cr 2p and I) B 1s spectra of CrB.

The morphology and microstructure of the as‐prepared CrB were first investigated by scanning electron microscopy (SEM). It is observed that the CrB delivers a nanometric lamination structure with a lateral size of ≈200–500 nm (**Figure** **1**A,B). The well‐defined edges and flat surfaces can be noticed, signifying a layered lattice structure. Transmission electron microscope (TEM) characterization was carried out for a more profound structural study as shown in Figure 1C. As expected, stacked layers can be noted with some exfoliated nanosheets scattering around, likely due to their peeled‐off upon the ultrasonic process, which strongly indicates the 2D lattice structure. The corresponding selected area electron diffraction (SAED) pattern is depicted in Figure 1D. The as‐developed CrB exhibits a somewhat single‐crystalline character, showing bright spots in an ordered arrangement. The marked spots can be assigned to the (111), (110), and (130) crystal faces of the orthorhombic CrB, respectively. Meanwhile, the high‐resolution TEM (HRTEM) and inverse fast Fourier transform (IFFT) reveal an explicit interplanar space of 0.277 nm assigned to the (110) facet of CrB (Figure 1E; Figure S1, Supporting Information), while the high‐angle annular dark field (HAADF) scanning transmission electron microscopy (STEM) image and energy dispersive X‐ray spectroscopy (EDS) mapping in Figure 1F confirm the uniform distribution of Cr and B in the layered product. In addition, the crystallographic structure of the as‐developed CrB was further confirmed by X‐Ray Diffraction (XRD, Figure 1G). The resulting pattern shows sharp peaks at 32.3, 38.3, 45.0, and 46.2 degrees corresponding to the (110), (021), (111), and (130) facets, respectively, of the orthorhombic CrB in Cmcm space group (PDF# 32–0277), which is well consistent with the SAED and HRTEM results. Therefore, it is reasonable to conclude that well‐crystallized CrB was successfully obtained in a typical 2D lattice structure, where the slabs of face‐sharing Cr_6_B trigonal prisms extend along the (010) plane and stack in the b‐direction, and the boron atoms are covalently bonded with each other to form zigzag chains parallel to the (001) direction. What should be further noted is that the interstitially alloyed structure with well‐inherited metal bonds in the matrix endows CrB with good metallicity and high electrical conductivity that significantly outperforms its oxide counterpart (357 vs 4.5 × 10^−3^ S m^−1^, Figure S2, Supporting Information). Beyond that, the coexistence of Cr─Cr (metallic), B─B (covalent), and Cr─B (ionic) bonds could trigger unique chemical features for potential host‐guest interactions with LiPS and thus regulates the sulfur electrochemical behaviors.

Given this, the surface chemistry of the obtained CrB was studied by X‐ray photoelectron spectroscopy (XPS). All the spectra were calibrated according to the C─C peak position of 284.8 eV (Figure S3, Supporting Information). Figure 1H depicts the Cr 2p spectrum of CrB, which witnesses three subpeaks at 574.1, 575.6, and 577.1 eV ascribed to the Cr^0^, Cr^3+^, and Cr hydroxide species, respectively.^[^ ^17^ ^]^ The strong Cr^0^ signal confirms the metallic character of CrB, while the rest species suggest the ionic feature as well as the potential partial oxidization/hydroxidization at the surface. Meanwhile, the B 1s spectrum exhibits characteristic peaks at 187.5 and 188.5 eV assigned to the B─Cr and B─B, respectively (Figure 1I),^[^ ^18^ ^]^ manifesting the coexistence of local ionic and covalent features in the CrB lattice. Oxidized B species such as B_2_O_3_ also exist, which is commonly observed in the literature.^[^ ^19^ ^]^ These results confirm the successful acquisition of CrB based on mild‐temperature thermal synthesis. The alloy structure well inherits the metallic character that would greatly benefit the electron conduction, while the as‐established local chemical polarity is expected to impose favorable chemical affinity to LiPS for achieving reliable sulfur immobilization and catalyzation.

**Figure 2:**
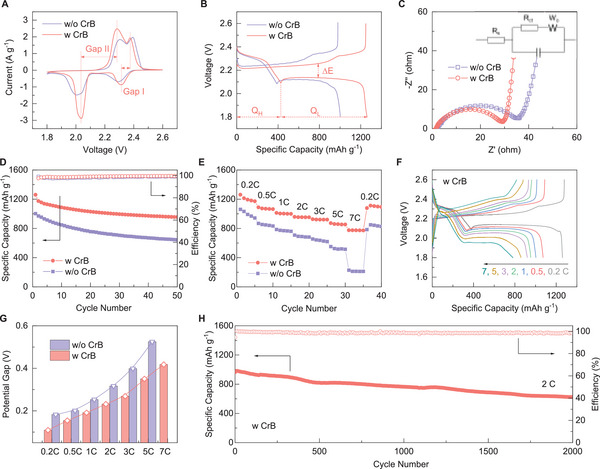
A) CV curves, B) charge–discharge profiles, C) Nyquist plots and equivalent circuit (inset), D) cycling performance at 0.2 C, E) rate performance of Li–S cells with or without CrB catalyst; F) charge–discharge of CrB cell at different rates; G) charge–discharge potential gaps of cells at different rates; H) long‐term cycling performance of CrB cell at 2 C.

In view of these potential benefits, the Li–S battery performance based on the CrB nanocatalyst was evaluated in coin cells (see details in Experimental Section). The CrB dosage in the electrodes was first optimized to 10 wt.% as shown in Figure S4 (Supporting Information). Skimpy addition seems hardly fulfill the catalytic advantages of CrB, while excessive CrB likely densifies the electrode with potential structural nonuniformity and thus deteriorates the electrochemical performance. **Figure** **2**A shows the CV curves of the cell based on the CrB catalyst (denoted as w CrB), while the one based on super P (SP) was employed as a contrast sample (denoted as w/o CrB). Distinctly, the CrB cell exhibits a much higher current response with a much sharper peak shape and smaller redox overpotential gap compared with those of the SP cell (61 vs 99 mV for Gap I, 248 vs 293 mV for Gap II), suggesting the considerably enhanced conversion kinetics by the CrB catalyst. This advance can also be confirmed by the Tafel plots as shown in Figure S5 (Supporting Information), the redox peaks referring to the Li_2_S conversion, which forms the Gap II, are specifically studied as it is generally considered the most kinetic‐limiting process in sulfur electrochemistry. During both the cathodic and anodic scanning, the CrB cell shows a much smaller Tafel slope (−33.4 and 65.1 mV dec^−1^) compared with those without the CrB catalyst (−67.3 and 71.3 mV dec^−1^), indicating the lowered energy barrier and facilitated Li_2_S redox by the CrB catalyst.

Correspondingly, the galvanostatic charge‐discharge profiles (Figure 2B) of the CrB cell demonstrate a much higher capacity (1260.9 vs 1002.7 mAh g^−1^) and smaller charge–discharge potential hysteresis (denoted as ∆E) compared with its SP counterpart (121 vs 165 mV, Figure S6, Supporting Information). Apart from that, the capacity ratio of the low‐potential plateau to the high‐potential plateau in the discharge curve (Q_L_/Q_H_) is another critical indicator of the sulfur redox efficiency. The much higher Q_L_/Q_H_ (1.975) of the CrB cell over its w/o CrB counterpart (1.594) illustrates that the sulfur species dissolved during the high‐plateau discharging can be reutilized and precipitated back on the cathode surface upon the low‐plateau stage. These results consistently and collectively demonstrate the much faster and more efficient sulfur redox behaviors on the CrB surface.

The kinetic improvement can also be perceived from the EIS analysis. As shown in Figure 2C, the Nyquist plots of both cells consist of a suppressed semicircle at the high‐medium frequency range, which is assigned to the charge‐transfer resistance (R_ct_), as well as an oblique line at the low‐frequency range referring to the Warburg resistance. The inset depicts the corresponding equivalent circuit. It can be noticed that the CrB cell delivers a much lower R_ct_ compared with the w/o CrB cell, while the much higher slope of the oblique line suggests the faster Li^+^ diffusion on the CrB surface. These results confirm the more facile charge supplies in the CrB‐based configuration and support its faster kinetic behaviors.

The galvanostatic cycling behaviors of different cells at 0.2 C were compared in Figure 2D. The CrB cell retains a capacity of 954.7 mAh g^−1^ after 50 cycles, corresponding to a capacity retention of 75.7%. By contrast, the SP cell experiences much faster capacity decay, showing a capacity of 644.4 mAh g^−1^ at the 50^th^ cycle with a lower retention of 64.2%. Apart from that, the rate capability of different cells was also compared by cycling at varied current rates, as shown in Figure 2E. The CrB electrode retains a favorably high capacity of 775 mAh g^−1^ even under a high rate up to 7 C, which recovers to 1112.4 mAh g^−1^ as the applied current returns to 0.2 C. In comparison, the control cell undergoes fast capacity decaying along with the increase of current rate and retains only a capacity of 228.4 mAh g^−1^ at 7 C. Such a distinctive comparison demonstrates the excellent reaction kinetic realized by the CrB electrocatalyst, which significantly promotes the sulfur conversions toward a superior rate capability. This improvement can be more vividly evidenced by the evolution of charge–discharge curves upon the multi‐rate cycling. Figure 2F shows the voltage profiles of the CrB cell at different current rates. It is noticed that the potential gap between the charge and discharge plateaus is gradually expanded along the rate increase from 0.2 to 7 C, yet the two‐platform discharge profile can be maintained. Whereas for the control cell without CrB, the severe polarization deforms the charge–discharge profiles significantly, failing to maintain the two‐plateau profile at 7 C (Figure S7, Supporting Information). The potential gap evolutions along the current rate increase are summarized in Figure 2G. It reveals the persistently lower overpotential of the CrB cell at the whole rate range compared with its w/o CrB counterpart, strongly confirming its much superior kinetic properties.

Additionally, the cycling behaviors of cells based on CrB and Cr_2_O_3_ were compared (Figure S8, Supporting Information). The Cr_2_O_3_ cell shows higher capacity, stability, and rate capability than the w/o CrB cell but is inferior to the CrB cell. The electrochemical improvement by Cr_2_O_3_ could be attributed to the decent sulfur confinement as illustrated by the static adsorption test (Figure S9, Supporting Information), whereas the lack of conductivity and catalytic activity limit the sulfur redox efficiency.^[^ ^20^ ^]^ Such a comparison further highlights the balanced adsorption and catalyzation by the CrB design, which enables fast and reversible sulfur electrochemistry. Given these advantages, a long‐term cycling evaluation was performed for the CrB cell. As depicted in Figure 2H, a stable cycling behavior can be achieved with a minimum capacity fading rate of 0.0176% per cycle over a prolonged 2000 cycles at 2 C. Meanwhile, a high coulombic efficiency close to unity can also be sustained, indicating excellent sulfur redox reversibility. These electrochemical performances are highly competitive among those reported in recent literature (Table S1, Supporting Information), further illustrating the superiorities of the CrB catalyst design.

**Figure 3:**
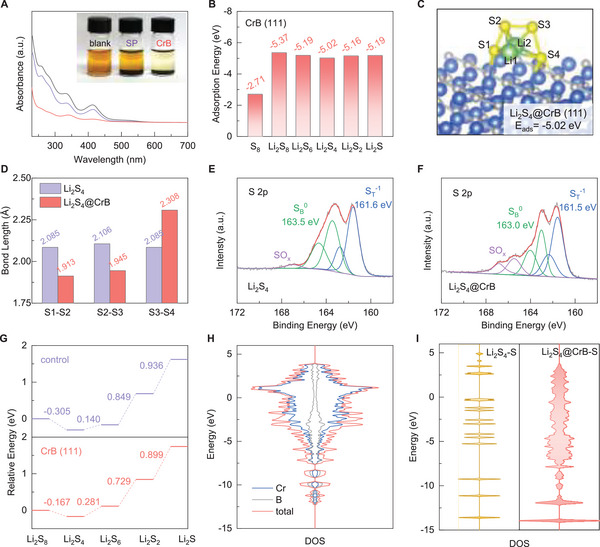
A) UV–vis spectra and optical images of polysulfide adsorbed by different samples; B) adsorption energies of various polysulfides on CrB (111) surface; C) geometrical configuration, D) bond lengths, XPS S 2p spectra of Li_2_S_4_ E) before and F) after adsorption on CrB; G) energy profiles of sulfur conversion with or without CrB catalyst; H) DOS pattern of CrB; I) DOS pattern of sulfur in Li_2_S_4_ before and after adsorption.

To understand these electrochemical improvements, the chemical interaction between the CrB catalyst and LiPS was studied first by static adsorption test. As presented in the inset of **Figure** **3**A, the blank Li_2_S_4_/tetrahydrofuran (THF) solution shows a typical brownish color. When immersed with the same amount of SP and CrB and let stand for 12 h, the solution with SP turns slightly lighter, while the one with CrB undergoes a strong decoloration, resulting in an almost transparent and colorless supernatant. Such a contrastive comparison strongly confirms the much superior LiPS adsorbability of CrB, which can be further verified by the lowest UV–vis absorbance of its corresponding supernatant (Figure 3A). Further probe into the interactive mechanism was carried out by DFT calculation. The CrB (111) facet was selected as a typical platform for the DFT study as it is the strongest exposed facet in the XRD result. A variety of sulfur species were simulated anchoring on the CrB (111) surface, contributing to adsorption energies of −2.71, −5.37, −5.19, −5.02, −5.16, and −5.19 eV, respectively (Figure 3B). The corresponding geometrically stable configurations are presented in Figure S10 (Supporting Information). These values are among the relatively high level in literature based on conventional carbon or metal compound substrates,^[^ ^21^ ^]^ confirming the strong chemical interactions between CrB and LiPS. Figure 3C shows specifically the geometry of Li_2_S_4_ adsorption on CrB. Interestingly, both chromium and boron are available and preferential to bond with terminal sulfur, forming a Cr─S─B bridging structure. This distinguishes from what is commonly observed in conventional metal compounds scenarios, such as oxides and sulfide, where the Lewis acid‐base principle governs the interactions with one end (e.g., metal sites) sulphophilic and the other (e.g., oxygen or sulfur sites) lithiophilic.^[^ ^22^ ^]^ The bond lengths of Cr─S are 2.333 and 2.409 Å, while the B─S lengths are 1.819 and 1.950 Å, suggesting a strong bonding strength. Such a dual sulfur‐bonding mode contributes to favorably high adsorption energy of −5.02 eV, while the lessened bondage with lithium may facilitate the ion transfer, as demonstrated above. Beyond that, a considerable distortion of polysulfide geometry can be noticed upon the interaction. As shown in Figure 3D and Figure S11 (Supporting Information), the bond lengths of S1─S2 and S2─S3 decrease significantly, accompanied by the drastic increase of the S3─S4 bond, referring to the compression of the Li_2_S_4_ molecule at one side and the stretch at the other. Such a distortion is also supported by the consistent variations of the Li─S bonds (Figure S12, Supporting Information). As a result, the lengthened S─S bond tends to be more easily cleaved, contributing to the facilitated conversion kinetics.

Furthermore, the interactive behaviors between CrB and polysulfide were analyzed by XPS. Figure 3E,F shows the S 2p XPS spectra of Li_2_S_4_ before and after the adsorption. Typically, the bare Li_2_S_4_ exhibits two pairs of peaks assigned to the terminal (S_T_^−1^, 161.6 eV) and bridging sulfur (S_B_^0^, 163.5 eV), respectively.^[^ ^23^ ^]^ A small peak can be detected at a relatively higher binding energy (BE), which could be ascribed to the slight oxidization upon the sampling. By contrast, the adsorption of Li_2_S_4_ on the CrB surface witnesses the peak shift to lower BE, particularly for the S_B_^0^ species, suggesting the electron‐accepting behavior of sulfur that increases the electron density and lowers the valence state. This well accords with the lengthened S─S bond as well as the formation of Cr─S bonds in the DFT result. Accordingly, the Cr 2p and B 1s XPS spectra were also compared. As shown in Figure S13A,B (Supporting Information), the Cr 2p XPS signal becomes drastically weaker after the adsorption, likely due to the strong polysulfide adsorption and coverage thereon. More importantly, the relative content of Cr (III) species significantly increases after the adsorption, confirming the establishment of the Cr─S bonding that oxidizes Cr and reduces S. The decrease of Cr hydroxide is likely due to its consumption by LiPS to form the sulfate‐related species, which is consistent with the S 2p spectrum. Meanwhile, a considerable peak shift to lower BE can be noticed in B 1s spectra (Figure S13C,D, Supporting Information), reflecting the electron transfer from sulfur to the electron‐deficient boron upon the B─S bonding. This behavior seems mainly occur at the terminal sulfur with higher electron density and partially offset the electron gained from Cr, thus resulting in the limited peak shift for S_T_^−1^. Overall, the XPS results are highly consistent with the DFT calculation, collectively confirming the strong and unique chemical interactions between CrB and polysulfides.

Given these interesting interactions, the kinetic behaviors of sulfur conversions on the CrB surface were studied as depicted in Figure 3G. The energy profile for bare sulfur conversion was also calculated as a control. It can be recognized that the rate‐determining step for sulfur conversions is the redox of Li_2_S in both cases, which is consistent with that commonly and experimentally observed. Notably, the CrB catalyst effectively lowers the energy barrier for Li_2_S redox from 0.936 to 0.899 eV. Meanwhile, a significantly lower energy barrier of 0.729 eV (vs 0.849 for the control) can be also achieved for another kinetic‐limiting step from Li_2_S_4_ to Li_2_S_2_. These results strongly illustrate the substantial promotion of sulfur conversion kinetics by the highly catalytic CrB. The density of states (DOS) of CrB was further calculated to unveil the underlying mechanism (Figure 3H). Strong and broad DOS can be observed around the Femi level, which is mainly contributed by the 3d electrons of Cr, indicating its excellent metallic character for rapid electron conduction. By contrast, the S pDOS of Li_2_S_4_ shows a discrete and cleaved pattern with several isolated bands along the energy axis and no distribution at the Fermi level (Figure 3I; Figure S14, Supporting Information), which well accords with its insulating nature. Interestingly, after the adsorption, the S pDOS converts into a continuous pattern with consideration distribution near the Femi level (Figure 3I). Such a drastic change in the S pDOS pattern strongly indicates that the adsorption on CrB provides sulfur with significantly enhanced electron transfer for fast reaction kinetics. Apart from that, strong overlapping between S and Cr as well as between S and B pDOS patterns can be witnessed (Figure S15, Supporting Information), further testifying the dual‐site interaction via strong S─Cr (p‐d) and S─B (p‐p) hybridizations that expedite the charge transfer for fast sulfur electrochemistry.

**Figure 4:**
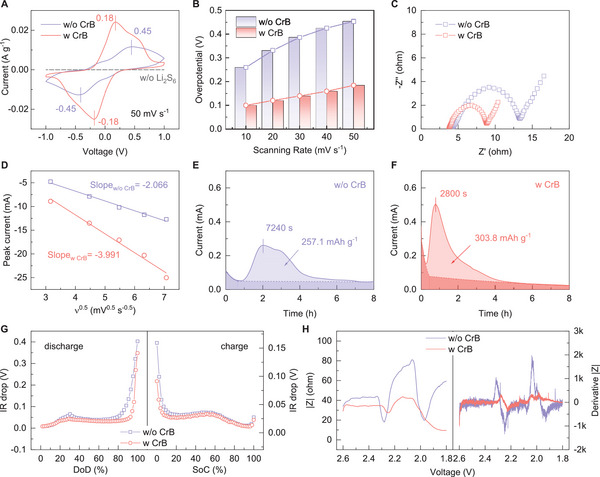
A) CV profiles, B) overpotentials at varied scanning rates, C) Nyquist plots of symmetric cells with and without CrB; D) Linear fitting of CV peak current versus square root of scanning rate; Li_2_S precipitation profiles on electrode E) without and F) with CrB; G) IR drops in GITT profiles, H) operando resistances and their first‐derivative of Li–S cells with or without CrB.

Inspired by the above computational results, the practical catalytic effect of CrB was further investigated by symmetric cell characterizations. **Figure** **4**A shows the CV profiles of polysulfide conversions at a scanning rate of 50 mV s^−1^ based on symmetric electrodes with or without the CrB catalyst. As expected, the electrode shows undetectable redox peaks without LiPS as the active species. Notably, the CrB catalyst enables a much higher current response with narrowed redox polarization compared with that without CrB (0.18 vs 0.45 V), manifesting the promoted polysulfide conversion on the CrB surface. In addition, CV curves at varied scanning rates were also recorded. The profile is well maintained upon the increase of scanning rate for the CrB cell, while serious profile deformation can be observed for that without CrB (Figure S16, Supporting Information). This can be more quantitively comprehended via the overpotential comparison as depicted in Figure 4B, where CrB enables much lower overpotential as well as its slower growth along the increase of scanning rate. Such a distinct comparison further confirms the significant kinetic promotion by the highly catalytic CrB, which can also be supported by the much smaller internal resistance of the CrB‐based symmetric cell (Figure 4C). Beyond that, the Li^+^ diffusion property is also a critical factor governing kinetic behaviors. Based on the Randles‐Sevcik equation, the symmetric cells' peak currents versus the scanning rate's square root were fitted, as shown in Figure 4D. It is noticed that the CrB cell delivers a more significant slope (−3.991 vs −2.066), indicating its higher Li^+^ diffusion coefficient that facilitates sulfur conversions.

In addition to the polysulfide redox, the liquid‐solid Li_2_S precipitation, which is considered the most kinetic‐limiting process, was specifically studied via the potentiostatic measurement. As shown in Figure 4E,F, the potentiostatic LiPS reduction initiates a current downslope followed by an upward peak, and gradually approaches the current limit after 8 h. The downslope and level‐off current (dark region) correspond to the reduction of residue Li_2_S_8_ and Li_2_S_6,_ respectively, while the left‐over upward peak part (light region) can be assigned to the Li_2_S formation and deposition.^[^ ^24^ ^]^ By comparison, the CrB catalyst enables a much earlier peak position (2800 vs 7240 s) and sharper peak shape compared with its counterpart, suggesting a lower energy barrier for Li_2_S nucleation and accumulation growth. As such, the CrB electrode obtains a higher precipitation capacity than that without CrB (303.8 vs 257.1 mAh g^−1^). Such an improvement can be also verified by the more uniform and smoother Li_2_S precipitation on the CrB‐loaded carbon paper compared with that without CrB (Figure S17, Supporting Information). Based on these specific studies, Galvanostatic Intermittent Titration Technique (GITT) and operando impedance tests further probed the overall sulfur kinetic behaviors. Figure S18 (Supporting Information) shows the GITT profiles of cells with and without the CrB catalyst. The corresponding IR drop evolutions upon the charge–discharge process were compared in Figure 4G. The IR drop refers to the gap between working potential and equilibrium potential at certain reaction status, which reflects the dynamic polarization upon the cell charge and discharge process. It is worth noting that the CrB cell shows persistently lower IR drop along the whole discharge–charge process, strongly confirming the alleviated polarization and the enhanced reaction kinetics by the CrB catalyst.

Furthermore, the operando resistance profiles were recorded for different cells as shown in Figure 4H. Upon the voltammetric scanning from 2.6 to 1.8 V at 0.2 mV s^−1^, both the cells begin with a fairly steady resistance and encounter a sudden drop at ≈2.3 V, which results from the initiation of polysulfide dissolution that expose the conductive surfaces. After that, a steep resistance rise can be noted at ≈2.2 V, owing to the polysulfide accumulation that increases the viscosity and deteriorates the ion transportation. Further cathodic scanning witnesses the drastic decline of resistance at ≈2.1 V, where Li_2_S starts to precipitate, accompanied by the recovery of electrolyte conductivity. Interestingly, at the end section of discharge, the CrB cell shows a continuous decrease of resistance, while the control cell undergoes a re‐rise of resistance, likely due to the electrode surface passivation by Li_2_S. This contrast further suggests that the CrB could regulate the Li_2_S precipitation behavior that facilitates the reaction kinetics. Apart from that, the operando profile of the CrB cell exhibits persistently lower resistance upon the discharging process compared with the w/o CrB counterpart. The first derivation of the profile more intuitively distinguishes the hysteretic and steadier resistance fluctuation for the CrB‐based configuration. Based on these results, it is reasonable to conclude that the unique CrB catalyst delivers a great capability of lowering the electrochemical resistance and promoting sulfur conversion, which contributes to excellent cycling and rate performance.

**Figure 5:**
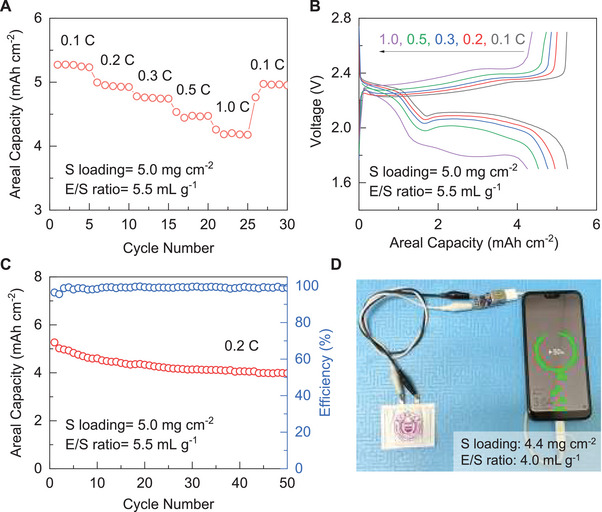
A) Rate performance, B) voltage profiles at different current rates, and C) cycling performance at 0.2 C of CrB cells under sulfur loading of 5 mg cm^−2^ and E/S ratio of 5.5 mL g^−1^; D) Optical image of CrB‐based Li–S pouch cell charging a cellphone.

Based on these encouraging superiorities, Li–S cells based on the CrB catalyst were further examined under high sulfur loading and limited electrolyte for potential practical application. **Figure** **5**A shows the rate performance of the CrB cell at a sulfur loading of 5.0 mg cm^−2^ and an electrolyte‐to‐sulfur (E/S) ratio of 5.5 mL g^−1^. An areal capacity of 5.27 mAh cm^−2^ can be obtained at 0.1 C, which retains at 4.53 mAh cm^−2^ even under a high rate of 1 C. What should also be noted is that the high‐loading CrB cell can basically maintain the two‐plateau discharging profile even under 1.0 C rate despite certain deformation (Figure 5B). These results strongly demonstrate the excellent reaction efficiency and kinetics realized by the CrB catalyst even under the high‐loading configuration. Moreover, the cycling performance was also evaluated. As shown in Figure 5C, a stable cycling behavior can be achieved by the CrB cell with a highly reversible capacity of 3.98 mAh cm^−2^ after 50 cycles at 0.2 C. Meanwhile, high coulombic efficiency close to unity can be obtained. These favorable results should be attributed to the strong sulfur adsorption and effective catalyzation of CrB, which inhibits the shuttle effect and promote the conversion reaction toward fast and durable sulfur electrochemistry. On this basis, the Li–S pouch cell with CrB electrode was further fabricated (Figure S19, Supporting Information), which is capable of successfully charging a smartphone, as well as powering electric clock and electric fan as depicted in Figure 5D and Figure S20 (Supporting Information), demonstrating an intriguing potential for practical application.

## 3 Conclusion

In summary, this work developed a unique CrB nanocatalyst for boosting sulfur electrochemistry. A well‐confined, mild‐temperature, solid‐state thermal method was designed to prepare CrB with lamination nanostructure. The highly conductive nature ensures a fast charge supply for electrochemical reactions, while more importantly, the dual‐sulphophilic feature of CrB establishes strong interactions with LiPS via simultaneous p‐d and p‐p hybridizations. Such a unique interaction imposes strong sulfur adsorption against the shuttle effect, and lowers energy barriers for multistep sulfur conversions, thus contributing to fast and efficient sulfur electrochemistry. As a result, Li–S cells based on CrB catalyst realize excellent cyclability over 2000 cycles, rate capability up to 7 C, and decent performance under high sulfur loading and limited electrolyte, demonstrating a good promise for the development of high‐performance and practically viable Li–S batteries.

## 4 Experimental Section

### Preparation of CrB

CrB was prepared via a confined solid thermal reaction. All the chemicals were used directly without further purification. Typically, 0.3 g chromium oxide (CrO_3_) and 2.0 g sodium borohydride (NaBH_4_) were mixed and sealed in a 20 mL stainless high‐pressure reactor under an Ar atmosphere, and then subjected to the thermal treatment at 500 °C for 10 h. After cooling, the product was washed with diluted hydrochloric acid to remove the by‐product and NaBH_4_ residue, and then washed repeatedly with deionized water and ethanol. The final CrB product was collected after vacuum drying for over 12 h.

### Preparation of Sulfur Electrodes

Sulfur electrodes were prepared by the conventional casting method. Typically, the slurry was prepared by dispersing elemental sulfur, SP, CrB, and PVDF with a weight ratio of 5:3:1:1 in NMP solvent. The homogeneous slurry was then cast onto the carbon‐coated aluminum foil and vacuum dried at 60 °C for over 12 h. The dried electrode was then cut into discs with a diameter of 12 mm for use. Sulfur loadings of 1.5 and 5.0 mg cm^−2^ were applied for regular and high‐loading electrode evaluations with the electrode thickness of 33 and 96 µm, respectively. Sulfur electrode containing elemental sulfur, SP, and PVDF in a weight ratio of 5:4:1 was also prepared for comparison.

### Electrochemical Evaluation

The electrochemical performance was evaluated in coin‐cell configuration. The cells were assembled using the prepared sulfur electrode as the cathode, Li foil as the anode, and Celgard 2400 membrane as the separator in an Ar‐filled glove box (O_2_<0.1 ppm, H_2_O<0.1 ppm). The electrolyte contains 1 m lithium bis(trifluoromethane)sulfonimide (LiTFSI) in the mixed solvent of 1,2‐dimethoxyethane (DME) and 1,3‐dioxolane (DOL) (1:1 in volume ratio) with 2 wt.% lithium nitrate (LiNO_3_) additive. The electrolyte‐to‐sulfur (E/S) ratio was controlled at 12 and 5.5 mL g^−1^, respectively, for regular and high‐loading configurations. The pouch cell was assembled using the CrB‐based sulfur cathode with a sulfur loading of 4.4 mg cm^−2^ and a thickness of 85 µm, lithium foil anode with a thickness of 50 µm, and a controlled E/S ratio of 4.0 mL g^−1^. Galvanostatic charge/discharge was performed by LAND testers (CT3002A). The current was calculated based on the mass of sulfur (1 C = 1675 mA g^−1^). The cyclic voltammetry of Li–S cells was recorded by CHI604E electrochemical workstation with a scanning rate of 0.1 mV s^−1^, while the electrochemical impedance spectra were collected within the frequency range of 0.1–100 k Hz under an amplitude of 5 mV.

### Polysulfide Adsorption Test

The Li_2_S_4_ solution (50 mM) was prepared by dissolving elemental sulfur and Li_2_S in a molar ratio of 3:1 in THF under vigorous stirring. After that, the same amount of absorbent, i.e., SP or CrB, was added to the as‐prepared homogenous Li_2_S_4_ solution and let stand for 12 h to observe the color variation.

### Kinetic Characterizations

The symmetric cells were fabricated by using identical electrodes as cathode and anode with an electrolyte containing 0.1 M Li_2_S_6_. The electrodes were prepared by loading the same amount (≈1 mg cm^−2^) of SP or CrB on carbon paper. The corresponding CV measurement was conducted at varied scanning rates from 10 to 50 mV s^−1^, while the EIS spectra were recorded as described above. Moreover, the Li_2_S deposition profiles were collected in asymmetric cells with CrB (or SP) cathode and Li foil anode. The Li_2_S_8_/tetraglyme (0.25 m) solution was specifically employed as the electrolyte according to the previous report.^[^ ^24^, ^25^ ^]^ The cells were galvanostatically discharged to 2.06 V and then subjected to potentiostatically discharge at 2.05 V for Li_2_S nucleation and precipitation until the current was lower than 10^−5^ A. In addition, operando resistance was recorded upon the voltammetric scanning from 2.6 to 1.8 V at a rate of 0.1 mV s^−1^. The applied amplitude was 5 mV, and the constant frequency was set at 15 Hz.

### Material Characterizations

The phase and crystalline characters were studied by X‐ray diffraction (XRD, Bruker‐AXS D8 Advances). Scanning electron microscopy (SEM, FEI Quanta 250F) and transmission electron microscopy (TEM, FEI Talos F200X G2) were applied to collect the morphological and microstructure information of the product. The chemical status was measured by X‐ray photoelectron spectroscopy (XPS, Thermo Scientific K‐Alpha). The UV–vis spectra were recorded on a UV‐3600 SHIMADZU spectrophotometer. The conductivities were obtained via the two‐electrode measurement. The CrB powder was sealed and densified in a plastic tube with tabs at each end connecting to the workstation. The conductivity (σ) was calculated based on the equation σ = L/(A·R), where L, A, and R refer to the length, sectional area, and measured resistance, respectively, of the sample cylinder.

### Theoretical Calculations

The Vienna Ab‐Initio Simulation Package (VASP) was employed for density functional theory (DFT) calculations.^[^ ^26^ ^]^ The Perdew‐Burke‐Ernzerhof (PBE) formulation was used within the generalized gradient approximation (GGA),^[^ ^27^ ^]^ while the projected augmented wave (PAW) potentials were applied to describe the ionic cores,^[^ ^28^ ^]^ The self‐consistency of electronic energy was determined by the energy change smaller than 10^−5^ eV. The geometry optimization was converged to the energy change smaller than 0.02 eV Å^−1^. The vacuum spacing perpendicular to the plane was set to be 18 Å. The weak interaction was described by DFT+D3 method using empirical correction in Grimme's scheme,^[^ ^29^ ^]^ Additionally, the Gibbs free energy was calculated based on the equation below:

(1) $$G\;=\;E_{\mathrm{elec}}\;+\;E_{\mathrm{ZPE}}-\;\mathrm{TS}$$

where E_elec_ refers to the electronic energy at 0 K calculated by DFT, E_ZPE_ is assigned to the zero‐point energy term. S and T are the entropy and the absolute temperature (i.e., 298.15 K in this case), respectively. The adsorption energies were determined as the Gibbs energy differences of the projected system before and after relaxation.

## Conflict of Interest

The authors declare no conflict of interest.

## Supporting information

Supporting Information

## Data Availability

The data that support the findings of this study are available on request from the corresponding author. The data are not publicly available due to privacy or ethical restrictions.
