# Supplementary material for: Dually Sulphophilic Chromium Boride Nanocatalyst Boosting Sulfur Conversion Kinetics Toward High‐Performance Lithium–Sulfur Batteries
Source: Adv Sci (Weinh). 2023 Sep 25;10(32):2303830. doi: 10.1002/advs.202303830 (PMC10646252; doi:10.1002/advs.202303830)
Supplement: Supplementary file 1 — Supporting Information [file ADVS-10-2303830-s001.pdf]

ADVANCED SCIENCE
Open Access

Supporting Information

for *Adv. Sci.*, DOI 10.1002/advs.202303830

Dually Sulphophilic Chromium Boride Nanocatalyst Boosting Sulfur Conversion Kinetics Toward High-Performance Lithium–Sulfur Batteries

Hongyang Li, Guxian Chen, Kailong Zhang, Liangbiao Wang and Gaoran Li**

Supporting information

Dually sulphophilic chromium boride nanocatalyst boosting sulfur conversion kinetics towards high-performance lithium-sulfur batteries

Hongyang Li, Guxian Chen, Kailong Zhang, Liangbiao Wang, Gaoran Li**

Hongyang Li, Guxian Chen, Gaoran Li
MIIT Key Laboratory of Advanced Display Materials and Devices
School of Materials Science and Engineering
Nanjing University of Science and Technology
Nanjing, Jiangsu 210094, P. R. China
E-mail: gaoranli@njust.edu.cn

Kailong Zhang
Key Laboratory for Palygorskite Science and Applied Technology of Jiangsu
National & Local Joint Engineering Research Center for Mineral Salt Deep Utilization
School of Chemical Engineering
Huaiyin Institute of Technology
Huaian, Jiangsu 223003, P. R. China.

Liangbiao Wang
School of Chemistry and Chemical Engineering
Jiangsu University of Technology
Changzhou Jiangsu 213001, P. R. China.
E-mail: lbwang@jsut.edu.cn

Supplementary Figures

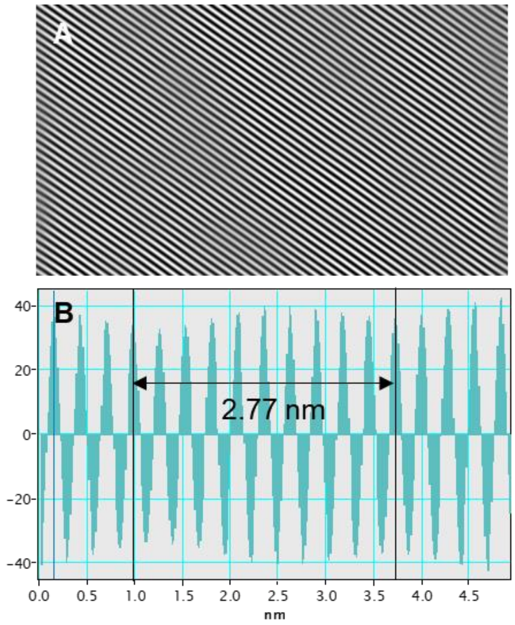

Figure S1. (A) IFFT pattern and (B) phase profile spectrum for (110) lattice orientation.

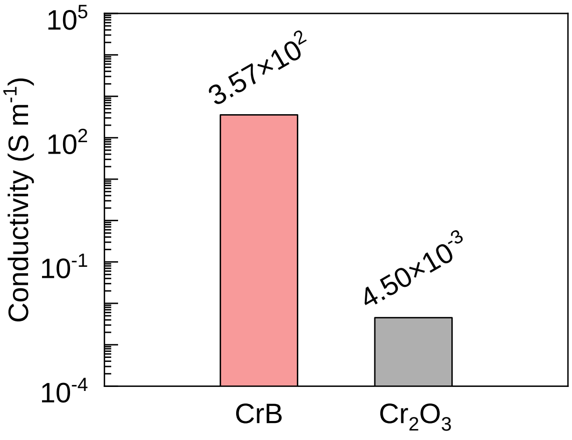

Figure S2. Conductivities of CrB and Cr_2O_3.

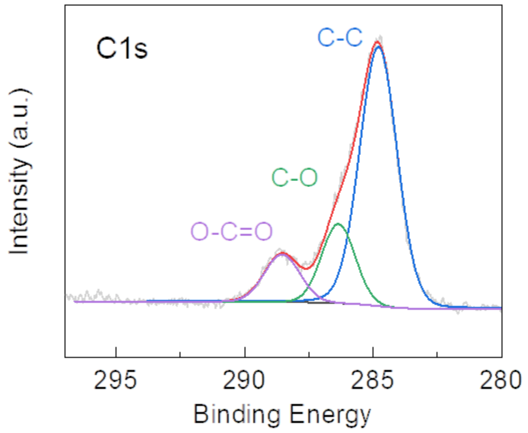

Figure S3. C1s spectrum of CrB.

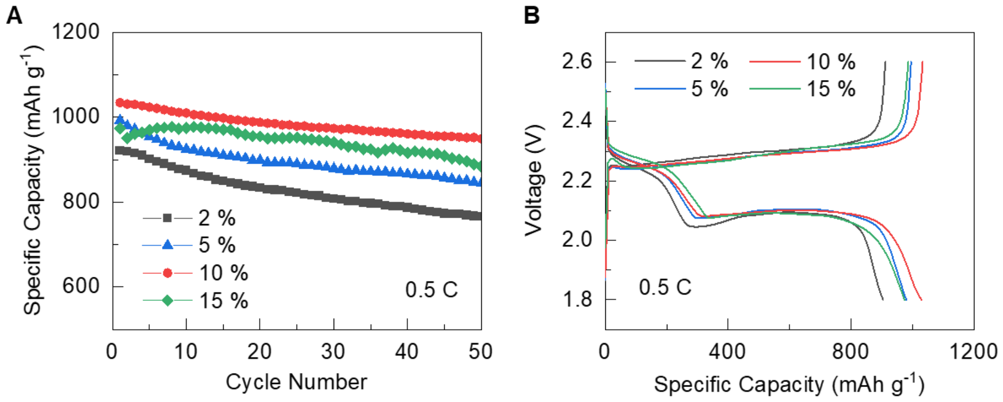

Figure S4. (A) Cycling performances and (B) voltage profiles of electrodes with different contents of CrB at 0.5 C.

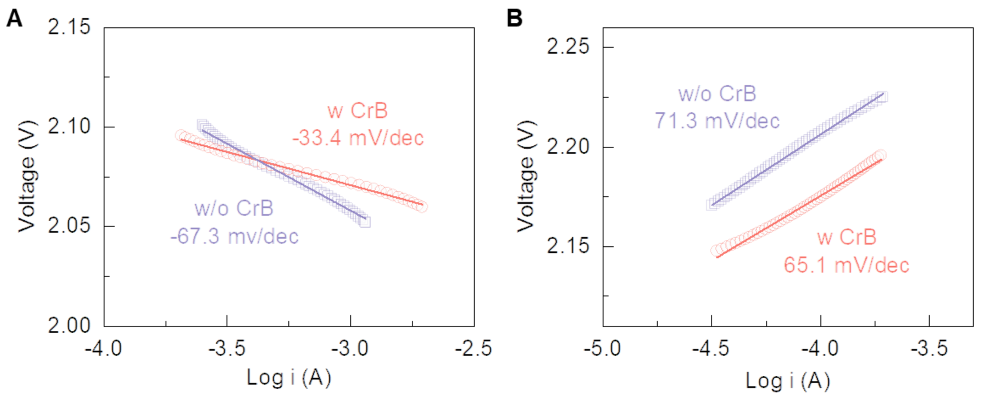

Figure S5. Tafel plots of the CV curves for different cells.

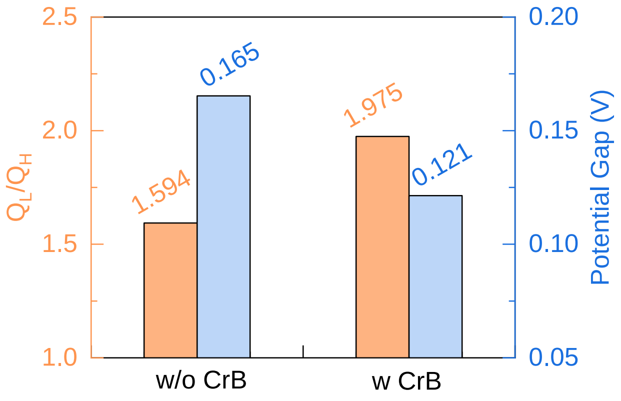

Figure S6. Q_L/Q_H values and potential gaps between charge and discharge curves in the voltage profiles of different cells.

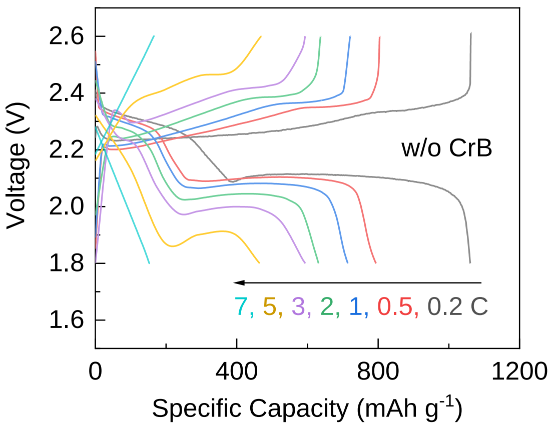

Figure S7. Charge-discharge profiles of the cell without CrB at different current rates.

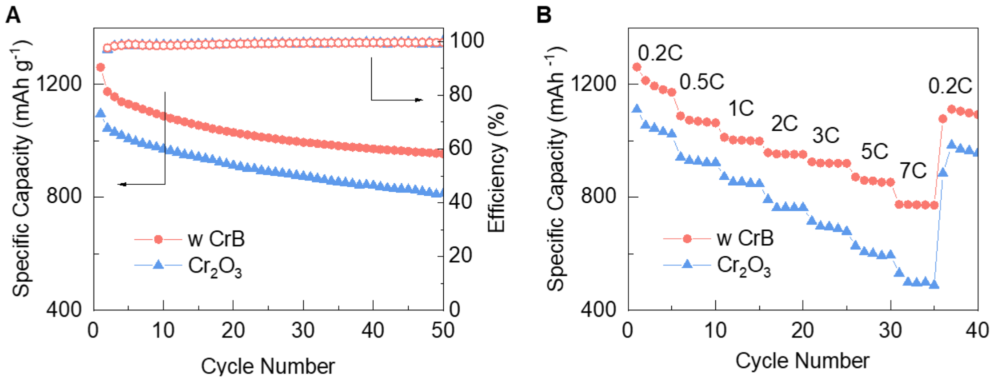

Figure S8. Comparisons of (A) cycling and (B) rate performances of CrB and Cr_2O_3 cells.

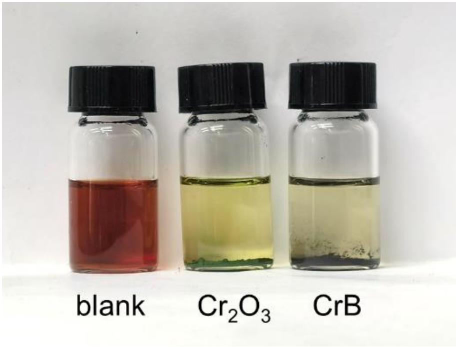

Figure S9. Polysulfide adsorption by Cr_2O_3 and CrB.

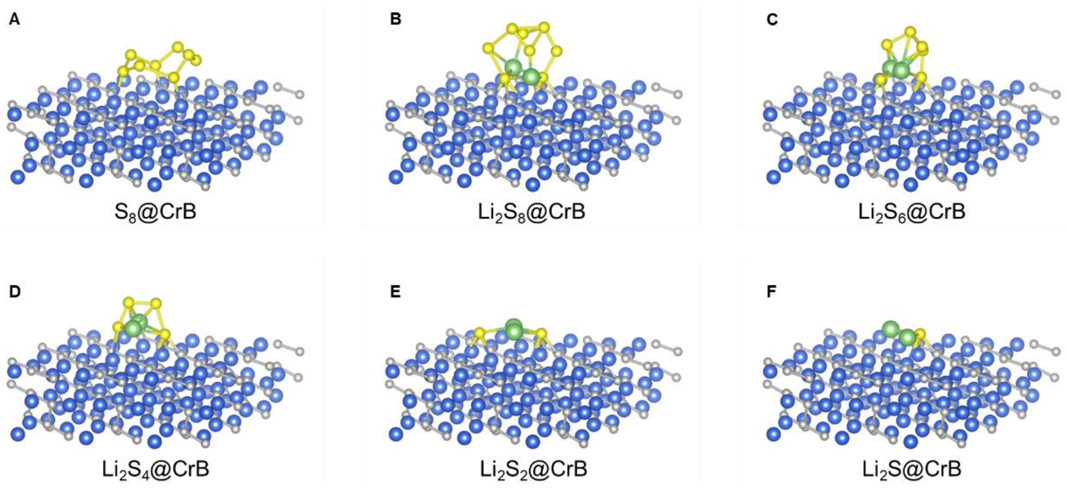

Figure S10. Geometrically stable configurations of different sulfur species on CrB surface.

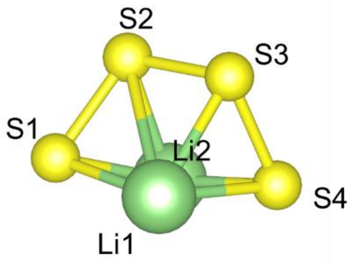

Figure S11. Stable geometry of Li_2S_4 cluster.

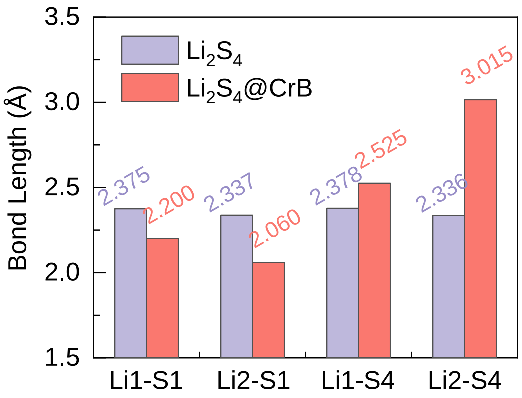

Figure S12. Li-S bond length variations upon the interaction between Li_2S_4 and CrB

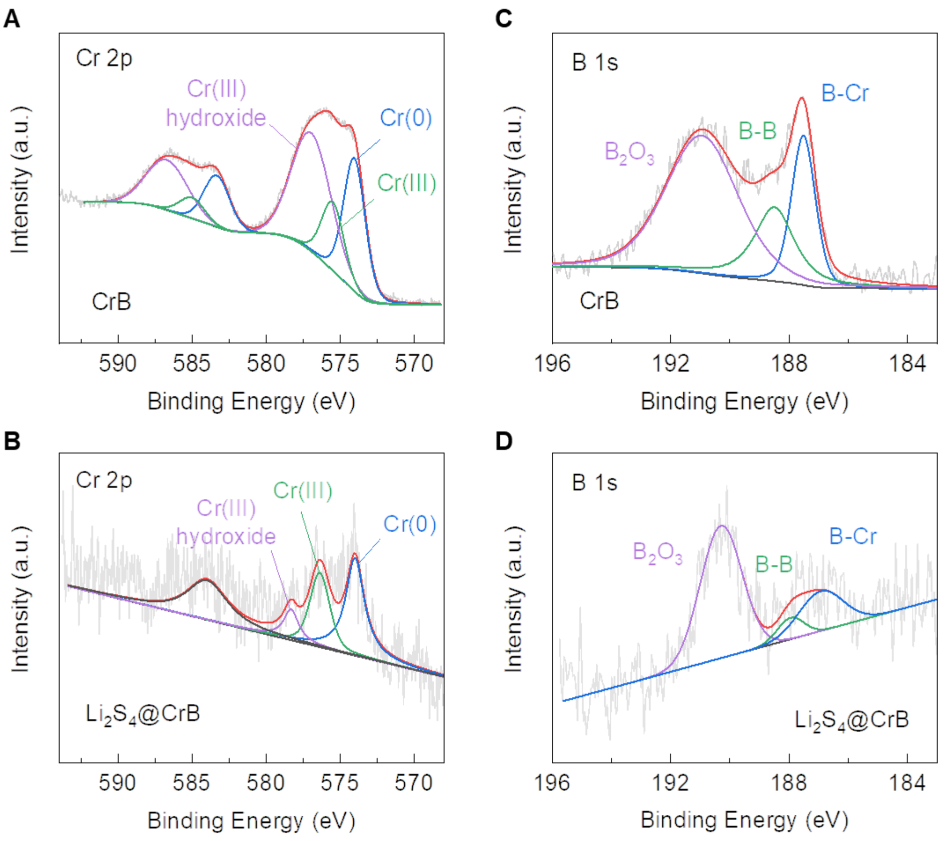

Figure S13. (A, B) Cr 2p and (C, D) B 1s spectra of CrB before and after Li_2S_4 adsorption

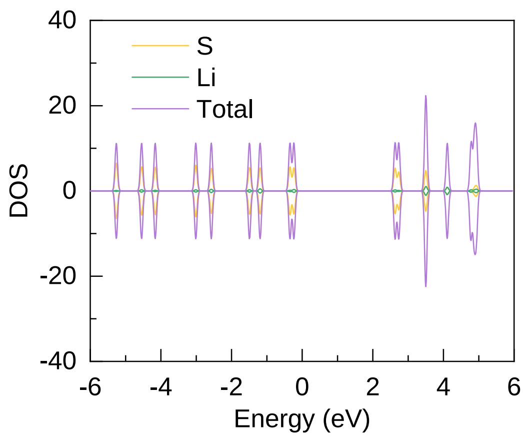

Figure S14. DOS pattern of Li_2S_4.

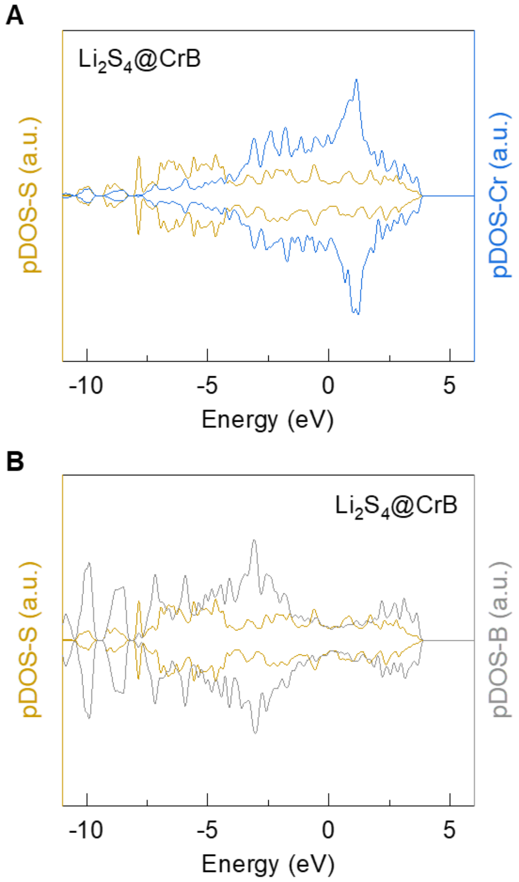

Figure S15. pDOS patterns of Li_2S_4@CrB composite.

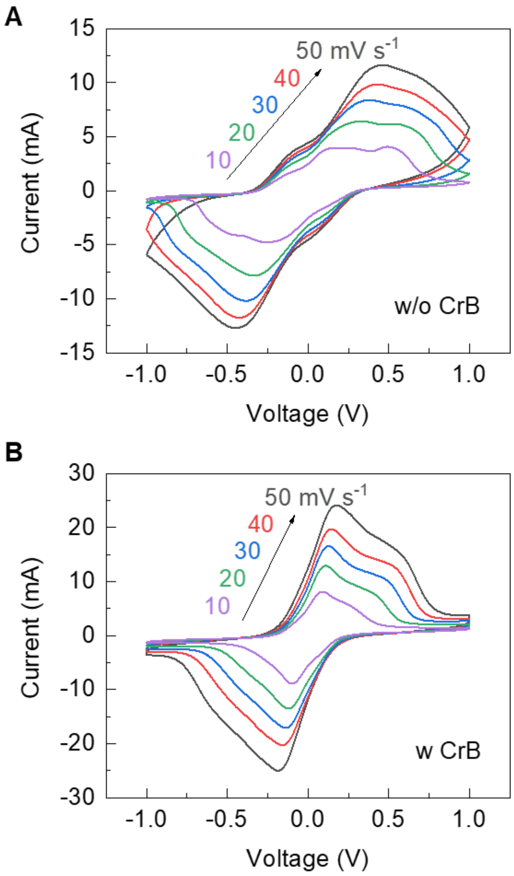

Figure S16. CV profiles of different symmetric cells at various scanning rates.

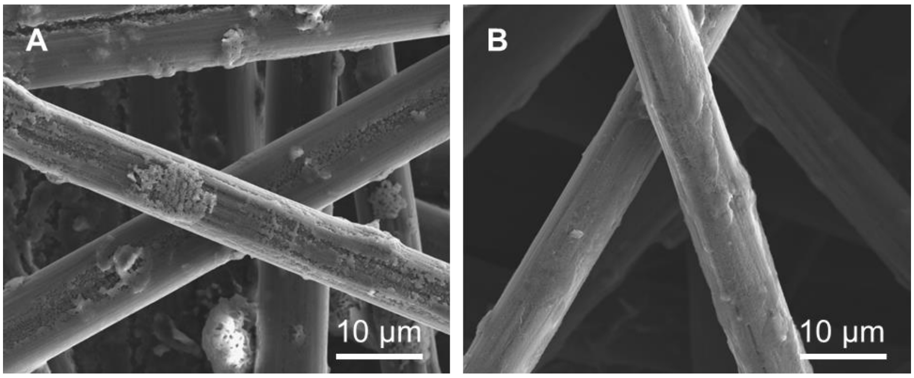

Figure S17. SEM images of Li_2S deposition on carbon paper with or without CrB.

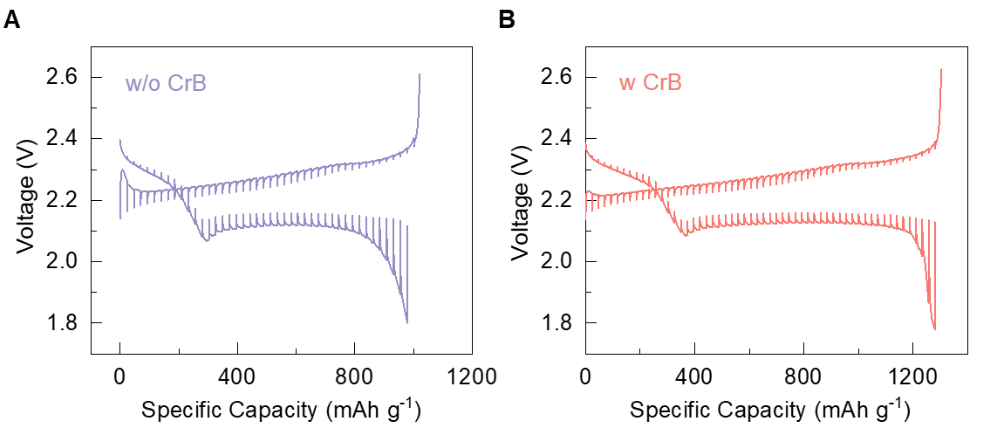

Figure S18. GITT profiles of cells (A) without and (B) with CrB catalyst.

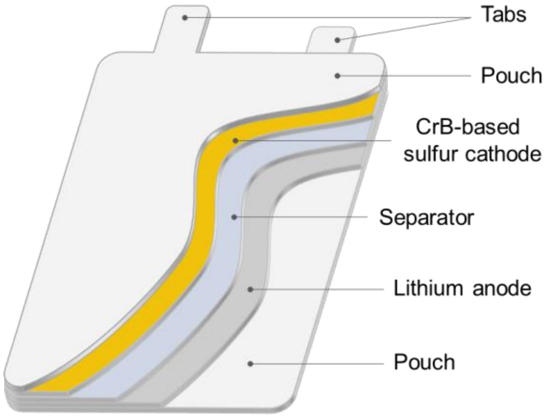

Figure S19. Scheme of the assembled pouch-cell configuration.

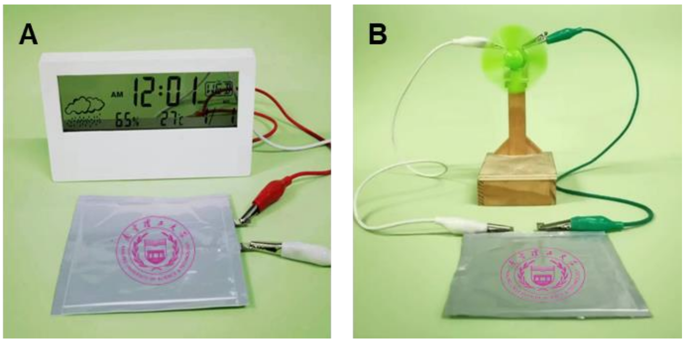

Figure S20. Li-S pouch cells based on CrB catalyst powering (A) electronic clock and (B) motor fan.

Supplementary Table

Table S1 Performance comparison among recent literature based on other metal boride catalysts

Sample	Rate Performance		Cycling Performance		High-loading Performance			Ref.
	Rate /C	Capacity /mAh g^{-1}	Cycle Number	Fading rate /%·cycle	S loading /mg cm^{-2}	E/S /mL g^{-1}	Capacity/ mAh cm^{-2}	
TiB_2	1	~800	500	0.0625	3.9	13	3.76	[1]
MoB	-	-	1000	0.03	6.1	7.0	4.75	[2]
NbB_2/NPG	10	609	1000	0.057	16.5	6.0	17	[3]
WB	-	-	1000	0.075	6.56	-	3.5	[4]
WB_2	2	730	500	0.05	3.8	10	4.4	[5]
CoB/G	2	850	450	0.029	2.3	20	3.55	[6]
CoB/MXene	5	597	2000	0.0088	5.1	-	5.2	[7]
NiB/CNT	2	750.5	500	0.07	8.3	3.0	9.24	[8]
ZrB_2/NG	2	869	600	0.056	8.03	8.0	8.08	[9]
Ni_3B/rGO	4	438.9	500	0.09	4.8	8.0	3.98	[10]
TiB_2/G	5	400	300	0.05	4.5	30	5.8	[11]
Mo-MoB	2	670.2	500	0.097	4.0	-	4.2	[12]
NbB_2/MXene	2	687.9	500	0.076	7.0	5.0	6.5	[13]
**CrB**	**7**	**775**	**2000**	**0.0176**	**5.0**	**5.5**	**5.27**	**This work**

Reference

[1] C. C. Li, X. B. Liu, L. Zhu, R. Z. Huang, M. W. Zhao, L. Q. Xu, Y. T. Qian, *Chem. Mater.* **2018**, *30*, 6969.

[2] R. J. He, A. Bhargav, A. Manthiram, *Adv. Mater.* **2020**, *32*, 2004741.

[3] B. Wang, L. Wang, B. Zhang, S. Y. Zeng, F. Tian, J. M. Dou, M. W. Zhao, Y. T. Qian, L. Q. Xu, *ACS Nano* **2022**, *16*, 4947.

[4] Y. W. Zhao, J. Li, J. L. Xiang, R. Wu, C. G. Lyu, H. F. Ma, X. F. Song, J. R. Zhang, L. Wang, C. Y. Zha, *Mater. Today Energy* **2022**, *25*, 100970.

[5] T. S. Sahu, V. Abhijitha, I. Pal, S. Sau, M. Gautam, B. R. Nanda, S. Mitra, *Small* **2022**, *18*, 2203222.

[6] B. Guan, L. S. Fan, X. Wu, P. X. Wang, Y. Qiu, M. X. Wang, Z. K. Guo, N. Q. Zhang, K. N. Sun, *J. Mater Chem. A* **2018**, *6*, 24045.

[7] B. Guan, X. Sun, Y. Zhang, X. Wu, Y. Qiu, M. X. Wang, L. S. Fan, N. Q. Zhang, *Chinese Chem. Lett.* **2021**, *32*, 2249.

[8] Z. Y. Wang, H. M. Wang, S. Liu, G. R. Li, X. P. Gao, *ACS Appl. Mater. Interfaces* **2021**, *13*, 20222.

[9] B. Wang, L. Wang, B. Zhang, Z. Kong, S. Y. Zeng, M. W. Zhao, Y. T. Qian, L. Q. Xu, *Energy Stor. Mater.* **2022**, *45*, 130.

[10] A. E. Shrshr, Y. T. Dong, M. A. A. Tahan, X. Y. Kang, H. Guan, X. F. Zheng, J. M. Zhang, *J. Alloys Compd.* **2022**, *910*, 164917.

[11] L. M. Jin, J. Ni, C. Shen, F. L. Peng, Q. Wu, D. H. Ye, J. S. Zheng, G. R. Li, C. M. Zhang, P. L. Zhou, *J. Power Sources* **2020**, *448*, 227336.

[12] Z. Guo, Y. Zhao, Y. Miao, D. Wang, D. Zhang, *ACS Appl. Energy Mater.* **2022**, *5*, 11844.

[13] D. Z. Lu, X. J. Wang, Y. J. Hu, L. G. Yue, Z. H. Shao, W. L. Zhou, L. Chen, W. Wang, Y. Y. Li, *Adv. Funct. Mater.* **2023**, 2212689.
